# The effect of maternal health service utilization in early initiation of breastfeeding among Nepalese mothers

**DOI:** 10.1186/s13006-019-0228-7

**Published:** 2019-07-31

**Authors:** Umesh Ghimire

**Affiliations:** New ERA, Kalopul, Rudramati Marga, Kathmandu, 44600 Nepal

**Keywords:** Early breastfeeding, Breastfeeding practice, Maternal health services utilization, Nepal

## Abstract

**Background:**

The World Health Organization (WHO) recommends early initiation of breastfeeding and exclusive breastfeeding for six months. Understanding the association of maternal health services and early initiation of breastfeeding might be useful on prioritizing the health services to promote early breastfeeding practices. The purpose of this study was to examine the association between utilization of maternal health services and early initiation of breastfeeding among Nepalese mothers.

**Methods:**

Nationally representative data from the 2016 Nepal Demographic Health Survey (NDHS) was used to determine the association between early initiation of breastfeeding and variables related to maternal health services utilization. Association was measured by using Chi-square test followed by calculation of adjusted odds ratio (AOR) and 95% confidence intervals (CI) using multivariable logistic regression analysis.

**Results:**

Out of 1,978 children, 55% were breastfed within an hour of birth. Early initiation of breastfeeding was associated among mothers who delivered at the health facilities (AOR 2.22; 95% CI 1.36, 3.60). Mothers who had a vaginal birth (AOR 6.70; 95% CI 4.30, 10.42) were significantly more likely to breastfeed within an hour of birth compared to mothers who had caesarean delivery. The odds of initiating early breastfeeding were higher among mothers from Province 5 (AOR 1.59; CI 1.02, 2.48), Province 6 (AOR 2.58; 95% CI 1.41,4.69) and Province 7 (AOR 2.30; CI 1.36, 3.87).

**Conclusions:**

Health facility delivery and a vaginal delivery were strongly associated with early initiation of breastfeeding. It is vital to intensify maternal health service up to the community to aware pregnant women to utilize maternal health services to improve breastfeeding practices. Skilled Birth Attendant (SBA) training should include comprehensive breastfeeding counselling package to motivate mothers to initiate early breastfeeding especially for mothers having caesarean delivery.

## Background

Annually, undernutrition causes 2.7 million child deaths in the world. One of the reasons for child undernutrition is due to a lack of optimal breastfeeding [1]. Early initiation of breastfeeding is the proportion of children born in the past 24 months who were put to the breast within an hour of birth [2]. The first breast milk after birth contains key nutrients required for an infant which provide energy and immunity to newborn [3]. Early breastfeeding is a behavior that also nurtures the first bonding between the mother and the baby [4]. Early initiation of breastfeeding together with exclusive breastfeeding during the first six months of life are the most significant ways to avert neonatal and infant mortality [5].

Globally, 50% of newborns are breastfed during the first hour of birth and only 40% of infants aged six months or less are exclusively breastfed [2, 6]. Despite cultural barriers in South Asian countries [7, 8], some improvements can be seen in the proportion of newborns receiving breast milk within an hour of birth [9–11]. Although Nepal’s progress in improving and maternal and child nutrition over the past decades was praiseworthy, utilization of maternal health services including the rate of early initiation of breastfeeding is still not at a satisfactory level [12]. Besides socioeconomic and cultural factors, studies have also reported that the underutilization of maternal health services such as antenatal care visit, institutional delivery, and postnatal care services, as key barriers in breastfeeding practices [13]. The main objective of this study was to assess the association of early initiation of breastfeeding and maternal health service utilization in Nepal. Findings of this paper are expected to provide further evidence to recommend interventions aimed at mothers, to utilize maternal health services and to recommend health facility-based practices to promote early breastfeeding practices in Nepal.

## Methods

Nationally representative data from the 2016 Nepal Demographic and Health Survey (NDHS) were used in this study. NDHS samples were selected in two stages in rural areas and three stages in urban areas. Stratified probability sampling design was employed to select the respondents. Wards were selected as a Primary Sampling Units (PSU) in rural areas and households were selected from the sampled PSU. In an urban setting, one Enumeration Area (EA) was selected from each PSU and then households were selected from the sample EAs.

The 2016 NDHS included 12,862 women between 15 and 49 years of age from 11,040 households [10]. The survey collected information on key demographic and health-related indicators such as family planning, fertility, mortality rates, maternal and child health status, and nutrition. In total, 5,038 mothers provided the information on breastfeeding for their last born children in the two years preceding the survey. The final sample for this study was selected from 1978 mothers who initiated breastfeeding their children within an hour of birth.

### Outcome variable

The early initiation of breastfeeding was defined by the WHO as the breastfeeding within the first hour of birth [2]. In the 2016 NDHS, mothers were asked about the duration of breastfeeding after childbirth and the response were recorded in hours if reported less than 24 h. For the analysis purpose, the response was categorized as i) “early initiation” if a child was put in the breast immediately or within one hour of birth and ii) “delayed breastfeeding” if a child was breastfed after one hour.

### Independent variables

Breastfeeding is influenced by various factors including demographic, biological, social, and psychological factors [14]. Several studies have used different models explaining the underlying factors associated with early initiation of breastfeeding. The selection and categorization of variables were done after a thorough review of literature from low middle-income countries [15–17].

The independent variables were categorized under three headings: i) socioeconomic characteristics of mother, ii) pregnancy-related characteristics, and iii) utilization of maternal health services by pregnant women. Socioeconomic characteristics included mother’s age, ethnicity, religion, wealth quintile, mother’s education, mother’s occupation, ecological region, province and place of residence. Pregnancy-related characteristics included mother’s age at first birth and birth order.

Frequency of ANC visits, place of delivery, delivery attended by a skilled health professional and mode of delivery were the variables that were included in the utilization of maternal health services by the mother. The NDHS collects information on assistance during delivery provided by all the trained health cadres but for the analysis purpose only doctor, nurse, and auxiliary nurse or midwives were included as the skilled health workers [10, 18].

Socioeconomic variables were categorized as per the need of analysis. The age of the respondent was categorized into five age groups: 15 to 19, 20 to 24, 25 to 29, 30 and above. The ethnicity of mother was categorized as per the caste classification adopted by further analysis series of the 2006 NDHS. It then was further grouped into four major caste categories: relatively advantaged (Brahmin/Chhetri), relatively disadvantaged (Janajati/Newar/Muslims), Disadvantaged (Dalits), and others (Madheshi and other unidentified) [19]. Other socioeconomic variables included ecological region, province and the place of residence. Education, the highest level of schooling attained by the mother was categorized as no education, primary, secondary, and higher. Occupation of the mother was measured as a categorical variable; not working, paid and agriculture. Birth order was classified into three groups: 1, 2 and 3 or more. Frequency of antenatal care visit was categorized as none, 1 to 3 and 4 and more ANC visits. Place of delivery was regrouped into delivery at the health facility and other. Assistance during delivery was categorized as assisted by skilled birth attendants (SBA) and other than SBA and the delivery mode was assessed as vaginal delivery and caesarean section.

## Data analysis

For this study, descriptive analysis was done to describe the background characteristics of mothers. Associations between the predictors and outcomes were first explored by using Pearson’s chi-square tests to determine whether early initiation of breastfeeding was statistically significant based on socioeconomic and maternal characteristics variables. Two models of multivariable logistics regression were used to estimate the adjusted odds ratio. In Model 1, variables related to maternal health service utilization (ANC visit, institutional delivery, and delivery attended by SBA) were analyzed adjusting all other background variables. The multivariable regression in Model 2 included only those variables that were found statistically significant in the Pearson’s chi-square test (*p* value < 0.05) in Table 2 together with variables related to maternal health service utilization. Variables with *p* value less than 0.05 were considered as statistically significant. To account the complex DHS sample design, all the analyses were performed using “svy” command and sampling weights were applied to adjust for design effects and the non-response rate. The analysis was performed using Stata version 15 [20].

## Results

Table 1 shows the general characteristics of mothers who were interviewed about breastfeeding practices after birth. Out of total 1,978 mothers, the majority (39%) were between 20 to 24 years of age. Relatively disadvantaged ethnicity represented the highest percentage while the poorest two quintiles had the lowest proportion. Most of the mothers were uneducated and unemployed. The highest number of women were from Province 2 and more than half of the mothers were from urban and Terai region. Slightly more than half of the mothers gave birth in adolescent age. More than two third of the mothers had received antenatal care services and 64 percent of mothers delivered their child in health facilities.

**Table 1:** General characteristics of mothers (weighted *n* = 1,978)

	*n* (%)
*Socioeconomic characteristics*	
Mother’s age (years)	
15–19	291 (14.70)
20–24	750 (37.91)
25–29	584 (29.52)
30 and above	354 (17.7)
Ethnicity	
Relatively advantaged	535 (27.1)
Relatively disadvantaged	767 (38.78)
Dalit	275 (13.9)
Others	401 (20.3)
Wealth quintile	
Poorest	414 (21.0)
Poorer	417 (21.1)
Middle	454 (23.0)
Richer	408 (20.6)
Richest	283 (14.4)
Mother’s education	
No education	570 (28.8)
Primary	391 (19.8)
Secondary	719 (36.3)
Higher	298 (15.1)
Mother’s occupation	
Not working	927 (46.9)
Paid	227 (11.5)
Agriculture	824 (41.6)
Place of residence	
Urban	1,062 (53.7)
Rural	916 (46.3)
Ecological region	
Mountain	131 (6.6)
Hill	760 (38.4)
Terai (plain land)	1087 (55.0)
Province	
Province 1	338 (17.1)
Province 2	513 (25.9)
Province 3	312 (15.8)
Province 4	164 (8.3)
Province 5	364 (8.3)
Province 6	121 (6.1)
Province 7	166 (8.4)
*Birth related characteristics*	
Mother’s age at first birth	
< 20	1027 (51.8)
> = 20	951 (48.1)
Birth order	
1	806 (40.7)
2	573 (28.9)
3 or more	600 (30.3)
*Characteristics related to maternal health service utilization*	
Frequency of antenatal visits	
None	72 (3.6)
1–3	505 (25.5)
4 and more	1401 (70.9)
Place of delivery	
Health facility	1270 (64.2)
Home	708 (35.8)
Professional assistance during delivery	
SBA	1277 (64.6)
Other than SBA	701 (35.4)
Mode of delivery	
Vaginal	1780 (90.0)
Caesarean	198 (10.0)

Out of 1978 mothers, 55 percent initiated breastfeeding their child within an hour of delivery (Table 2). Mother’s age category 20 to 24 years reported having the highest percentage (57.6%) of early breastfeeding. The proportion was less in terms of women belonging to Madhesi and other unidentified ethnic groups. Mother from the poorest wealth quintile and with secondary level education reported the highest proportion of early breastfeeding. Unemployed mothers had the lowest proportion (50.7%) of breastfeeding practice within an hour of delivery. In regard to the place of residence disaggregation, a higher proportion of mother from urban, Hill and Province 7 had breastfed their child within one hour of delivery.

**Table 2:** Distribution of mothers who started breastfeeding within an hour of birth according to the explanatory factors in Nepal (weighted *n* = 1,978)

	Initiation of breastfeeding		
	Early	Delayed	
	*n* (%)	*n* (%)	*p* value
Socioeconomic characteristics			
Mother’s age (years)			0.339
15–19	160 (55.0)	131 (45.0)	
20–24	432 (57.6)	318 (42.4)	
25–29	301 (51.6)	283 (48.4)	
30 and above	193 (54.6)	160 (45.4)	
Ethnicity			0.008
Relatively advantaged	325 (60.8)	210 (39.2)	
Relatively disadvantaged	423 (55.2)	344 (44.8)	
Dalit	155 (56.3)	120 (43.7)	
Others (Madheshi and other unidentified)	183 (45.7)	218 (54.3)	
Wealth quintile			0.094
Poorest	256 (61.9)	158 (38.1)	
Poorer	225 (53.8)	193 (46.2)	
Middle	232 (51.1)	222 (48.9)	
Richer	230 (56.2)	179 (43.8)	
Richest	144 (50.7)	140 (49.4)	
Mother’s education			0.071
No education	280 (49.1)	290 (50.9)	
Primary	222 (56.8)	169 (43.2)	
Secondary	421 (58.5)	298 (41.5)	
Higher	164 (55.0)	134 (45.0)	
Mother’s occupation			0.015
Not working	471 (50.7)	457 (49.3)	
Paid	128 (56.2)	99 (43.8)	
Agriculture	488 (59.3)	335 (40.7)	
Place of residence			0.144
Urban	606 (57.0)	456 (43.0)	
Rural	481 (52.5)	435 (47.5)	
Ecological region			0.033
Mountain	80 (61.3)	51 (38.7)	
Hill	444 (58.4)	316 (41.6)	
Terai (Plain land)	563 (51.7)	525 (48.3)	
Province			< 0.001
Province 1	175 (51.6)	164 (48.4)	
Province 2	232 (45.3)	281 (54.7)	
Province 3	176 (56.4)	136 (43.6)	
Province 4	90 (54.7)	74 (45.3)	
Province 5	215 (59.1)	149 (40.9)	
Province 6	82 (67.6)	39 (32.4)	
Province 7	117 (70.7)	49 (29.3)	
Pregnancy-related characteristics			
Mother’s age at first birth (years)			0.443
< 20	574 (55.9)	453 (44.1)	
> = 20	513 (53.9)	438 (46.1)	
Birth order			0.978
1	442 (54.8)	364 (45.2)	
2	317 (55.3)	256 (44.7)	
3 or more	328 (54.7)	272 (45.3)	
Characteristics related to maternal health service utilization			
Frequency of antenatal visits			0.118
No visit	36 (50.2)	36 (49.8)	
1–3	253 (50.1)	252 (49.9)	
4 and more	797 (56.9)	604 (43.1)	
Place of delivery			< 0.001
Health facility	753 (59.3)	517 (40.6)	
Home	333 (47.0)	375 (53.0)	
Professional assistance during delivery			0.003
SBA^a^	744 (58.3)	533 (41.7)	
Other than SBA	343 (48.9)	359 (51.1)	
Mode of delivery			< 0.001
Vaginal	738 (41.5)	1042 (58.5)	
Caesarean	153 (77.5)	45 (22.5)	

^a^ includes doctor, nurse and midwives

*p* value is based on the Pearson chi-square test

Early initiation of breastfeeding was found to be higher among women who had four or more ANC visits and who delivered their child in the health facilities. Likewise, deliveries attended by SBA (58%) had a better result in terms of breastfeeding within the first hour of childbirth, showing a significant association. Similarly, ethnicity, occupation of mother, place of delivery and province were significantly associated with early initiation of breastfeeding. However, no association was observed in term of frequency of ANC visits, and delivery assisted by SBA. Out of three variables selected for maternal health service utilization, two variables; place of delivery and professional assistance during delivery showed a significant association.

Independent variables with a statistically significant test in Table 2 were included in logistic regression Model 2. Table 3 shows the logistics regression models exploring the association of early initiation of breastfeeding. As reported in both logistics regression models, mothers who had delivered at health facilities (AOR 2.22, 95% CI 1.36, 3.60) were two times more likely to initiate the breastfeeding within an hour of birth. Delivery method had a positive effect on early initiation of breastfeeding, showing that mothers having a vaginal delivery (OR 6.70; 99% CI 4.30, 10.42) were more likely to initiate early breastfeeding than the mothers with a caesarean delivery. Compared to the mother in Province 2, mothers in Province 6 (AOR 2.58, 95% CI 1.41, 4.69), Province 7 (AOR 2.30, 95% CI 1.36, 3.87) and Province 5 (AOR 1.59, 95% CI 1.02, 2.48) were more likely to initiate early breastfeeding within an hour of delivery.

**Table 3:** Adjusted Odds Ratio (95% CI) estimates of effects of utilization of maternal health service and initiation of breastfeeding within an hour after birth in Nepal, 2016

Determinants	Model 1^a^		Model 2	
	AOR (95% CI)	*p* value	AOR (95% CI)	*p* value
Frequency of antenatal visits				
No visit (Ref)	1.00		1.00	
1–3	0.84 (0.47, 1.49)	0.55	0.94 (0.52, 1.68)	0.83
4 +	1.00 (0.56, 1.77)	0.99	1.03 (0.57, 1.87)	0.92
Place of delivery				
Home (Ref)	1.00		1.00	
Health facility	2.46 (1.53, 3.96)	< 0.001	2.22 (1.36, 3.60)	< 0.001
Professional assistance during delivery				
Others^b^ (Ref)	1.00		1.00	
SBA	0.84 (0.51, 1.38)	0.49	0.95 (0.57, 1.58)	0.84
Mode of delivery				
Caesarean (Ref)	1.00		1.00	
Vaginal	6.72 (4.42, 10.22)	< 0.001	6.70 (4.30, 10.42)	< 0.001
Ethnicity				
Relatively advantaged (Ref)			1.00	
Relatively disadvantaged			1.07 (0.78, 1.46)	0.68
Dalit			1.11 (0.76, 1.64)	0.59
Others			0.92 (0.58, 1.44)	0.55
Occupation of women				
Paid (Ref)			1.00	
Not working			0.84 (0.56, 1.25)	0.39
Agriculture			0.94 (0.64, 1.38)	0.75
Ecological zone				
Hill (Ref)			1.00	
Mountain			1.08 (0.67, 1.78)	0.74
Terai (plain land)			1.08 (0.75, 1.55)	0.66
Province				
Province 2 (Ref)			1.00	
Province 1			1.34 (0.81, 2.22)	0.25
Province 3			1.62 (0.93, 2.82)	0.09
Province 4			1.51 (0.87, 2.63)	0.15
Province 5			1.59 (1.02, 2.48)	0.04
Province 6			2.58 (1.41, 4.69)	< 0.001
Province 7			2.30 (1.36, 3.87)	< 0.001

*AOR* adjusted odds ratio, *CI* confidence interval, *Ref* Reference group

^a^ adjusted to ethnicity, occupation, ecological zone, province

^b^ includes doctor, nurse and midwives

## Discussion

The 2016 Nepal DHS reported that 54.9% of mother initiated breastfeeding their child within an hour of delivery. Association between early initiation of breastfeeding and factors other than utilization of maternal health services were explored in earlier studies [21, 22]. This study explores the effect of mother’s utilization of maternal health service with the early initiation of breastfeeding among Nepalese mothers. Findings of this study suggest that mothers who gave birth at the health facility were more likely to initiate early breastfeeding within an hour. This finding is consistent with the earlier studies conducted in Nepal, Ghana and Northwest Ethiopia [23–25]. Many studies have identified home delivery as a major barrier to early initiation of breastfeeding [13, 26]. Mothers of babies delivered in the health facilities can benefit from direct counseling and encouragement from health workers who promote a positive environment for early initiation of breastfeeding. Several studies done in various countries suggested that health facility-based breastfeeding promotional strategies have demonstrated improved breastfeeding practices in the hospital setting [27–30]. Antenatal appointments during pregnancy provide an exceptional opportunity for mothers to have counselling on breastfeeding practices [31]. No significant association was observed between the frequency of ANC visits and early initiation of breastfeeding in this study which is contrary to the previous studies [32, 33]. Professional assistance during delivery provided by the skilled health workers is a critical indicator for maternal and child survival [34]. The 2016 NDHS reported that 57% of the births were delivered in health facilities and this figure was increased by nearly 22% point compared to the 2011 NDHS [10].

Early initiation of breastfeeding was significantly associated among mothers who had a vaginal delivery. This finding was in agreement with the studies conducted in India, Sri Lanka, and Bangladesh that women who undergo caesarean delivery were less likely to initiate breastfeeding as compared to the women with vaginal birth [32, 35, 36]. After caesarean delivery, exhaustion and post-caesarean pain contribute to the delayed breastfeeding practice [37]. Studies suggested that caesarean deliveries were associated with delayed breastfeeding and further breastfeeding support are needed along with focused counseling and encouragement to the mothers in order to provide appropriate anticipatory guidance to reduce difficulties [38, 39].

The findings reveal that the mother’s province was found to be associated with the early initiation of breastfeeding. Mothers from Province 5, Province 6 and Province 7 were more likely to breastfeed early than the mothers in Province 2. Province 6 and Province 7 have the hard to reach terrains in Nepal with the lowest Human Development Index (HDI) [40]. Socioeconomic inequalities along with geographic constraint coexist in this part of the country that impedes service utilization by the mothers [41]. Nevertheless, urban areas where there is easy accessibility of commercial prelacteal feeding, the percentage of initial breastfeeding is shockingly low. In case of Province 6 and Province 7, factors such as low socioeconomic status of people, and inaccessibility of prelacteal feeds could have contributed to higher rate early initiation of breastfeeding [10, 42].

Although this study has identified factors that determine the association of early initiation of breastfeeding in Nepal, few limitations that it entail could not be ignored. Nepal DHS data being a cross-sectional in nature, cause-effect relation between outcome and predictors cannot be determined. Some important variables such as distance to the nearest health facility (other than government health facilities) and information on breastfeeding counseling by health workers that possibly have effect on maternal health services utilization could not be included in the analysis due to the limitation in dataset. The demographic and health survey only capture the doctor, nurse, and midwives as skilled birth attendants, while other large numbers of health cadres such as health assistant and auxiliary health workers who are trained on skilled birth attendant are excluded in the survey. Despite these limitations, the study has outlined some important findings on early initiation of breastfeeding which would be propitious in prioritizing maternal and child health program in Nepal.

## Conclusions

Mother’s attainment of maternal health services such as delivery at health facilities and vaginal delivery were found to have a positive association with early initiation of breastfeeding. It is important to extend maternal and child health program up to the community level and aware pregnant women to utilize maternal health services to improve breastfeeding practices. SBAs should be provided with comprehensive training on breastfeeding techniques and focused counselling to initiate breastfeeding as early as possible to the recently delivered mothers, particularly to the mothers having a caesarean delivery.

## Data Availability

Data of Nepal Demographic and Health Survey are freely available at dhsprogram.org after registering for the specific project.

## Abbreviations

ANC: Antenatal Care
CI: Confidence Interval
EA: Enumeration Area
HDI: Human Development Index
NDHS: Nepal Demographic and Health Survey
NHRC: Nepal Health Research Council
OR: Odds Ratio
PSUs: Primary Sampling Units
SBA: Skilled Birth Attendant
SDG: Sustainable Development Goal
WHO: World Health Organization
